# Healthcare seeking behaviour for common infectious syndromes among people in three administrative regions of Johannesburg, South Africa, 2015: a cross-sectional study

**DOI:** 10.11604/pamj.2019.33.159.18461

**Published:** 2019-07-03

**Authors:** Relebogile Mapuroma, Cheryl Cohen, Lazarus Kuonza, Alfred Musekiwa, Stefano Tempia, Akhona Tshangela, Claire von Mollendorf

**Affiliations:** 1 School of Health Systems and Public Health, Faculty of Health Sciences, University of Pretoria, Pretoria, South Africa; 2 South African Field Epidemiology Program, National Institute for Communicable Diseases of the National Health Laboratory Service, Johannesburg, South Africa; 3 Centre for Respiratory Diseases and Meningitis, National Institute for Communicable Diseases of the National Health Laboratory Service, Johannesburg, South Africa; 4 School of Public Health, Faculty of Health Sciences, University of the Witwatersrand, Johannesburg, South Africa; 5 Wits Reproductive Health & HIV Institute (Wits RHI), Faculty of Health Sciences, University of the Witwatersrand, Johannesburg, South Africa; 6 US Centers for Disease Control and Prevention, Pretoria, South Africa; 7 Influenza Division, Centers for Disease Control and Prevention, Atlanta, Georgia, USA; 8 MassGenics, Duluth, Georgia, USA

**Keywords:** Disease surveillance, healthcare utilisation, respiratory illness, infectious diseases, household survey, South Africa

## Abstract

**Introduction:**

Hospital-based surveillance programs only capture people presenting to facilities and may underestimate disease burden. We conducted a healthcare utilisation survey to characterise healthcare-seeking behaviour among people with common infectious syndromes in the catchment areas of two sentinel surveillance hospitals in Johannesburg, South Africa.

**Methods:**

A cross-sectional survey was conducted within three regions of Johannesburg from August to November 2015. Premises were randomly selected from an enumerated list with data collected on household demographics and selected syndromes using a structured questionnaire. Fisher's exact or chi-square tests were used to determine association of characteristics among different regions.

**Results:**

Of 3650 selected coordinates, 3358 were eligible dwellings and 2930 (87%) households with 9850 individuals participated. Four percent of participants (431/9850) reported influenza-like illness (ILI) in the last 30 days; equal numbers of participants (0.2%, 20/9850) reported pneumonia or tuberculosis symptoms in the last year and <1% reported diarrhoea or meningitis symptoms. Sixty eight percent (295/431) of participants who reported ILI, 75% (6/8) of children with diarrhoea and all participants who reported pneumonia (20), tuberculosis (20) or meningitis (6) sought healthcare. For all syndromes most sought care at registered healthcare providers. Of these only 10% (24/237) attended sentinel hospitals, predominantly those that lived closer to the hospitals. In contrast, of patients with meningitis, 50% (3/6) sought care at sentinel hospitals.

**Conclusion:**

Patterns of seeking healthcare differed by syndrome and distance from facilities. Surveillance programs are still relevant in collecting information on infectious syndromes and reflect a proportion of the hospital's catchment area.

## Introduction

In South Africa, tuberculosis, influenza and pneumonia were among the top ten causes of death in all age groups in 2014-2016 [1]. In 2016, of 456,612 total deaths reported, tuberculosis was responsible for 29,680 (6.5%), and pneumonia and influenza for 19,634 (4.3%) [1]. In children under 15 years of age, intestinal infectious diseases and central nervous system inflammatory diseases were in the top ten causes of deaths in 2016 [1]. Since 2009, the National Institute for Communicable Diseases (NICD) has conducted active, prospective, hospital-based surveillance for pneumonia in five of South Africa's nine provinces to describe its epidemiology, characterise causative pathogens and prepare for pandemics and outbreaks [2]. From 2012 the NICD conducted prospective surveillance for persons with influenza-like illness (ILI) at outpatient clinics located in the catchment areas of two pneumonia surveillance sites [3]. Standard World Health Organization case definitions were used for ILI and severe acute respiratory illness surveillance, with respiratory samples tested using a multiplex real-time reverse transcription-polymerase chain reaction [4]. The NICD also has active laboratory-based surveillance which monitors several pathogens including those causing diarrhoea and meningitis [5]. The disadvantage of facility-based surveillance programs is that they only capture information for people seeking healthcare at facilities conducting surveillance. This can lead to inaccurate interpretation of surveillance data as other people are missed [6]. South Africa has a health information system but this also captures information at a facility level. A healthcare utilisation survey (HUS) captures information on health seeking behaviour that is missed by surveillance programs [7]. Several HUSs have been conducted in different countries to complement surveillance program data [8-10]. As communities differ, it is important to understand healthcare seeking patterns in different settings [9]. Different individual and healthcare provider factors may influence healthcare seeking behaviour [11, 12]. Knowledge of healthcare utilisation behaviour within communities is useful when developing health policies and implementing health programs. The study objective was to describe healthcare seeking behaviour for individuals with common infectious syndromes in the catchment areas of two sentinel surveillance hospitals in Johannesburg, South Africa.

## Methods

**Study design:** we performed a cross-sectional community-based HUS using a one-stage cluster design, with households as the sampling unit, similar to previously described methods [9]. We collected data on three respiratory syndromes (tuberculosis, ILI and pneumonia), meningitis and diarrhoea in 27 suburbs in regions A, B and C of Johannesburg, South Africa from August to November 2015.

**Figure 1:**
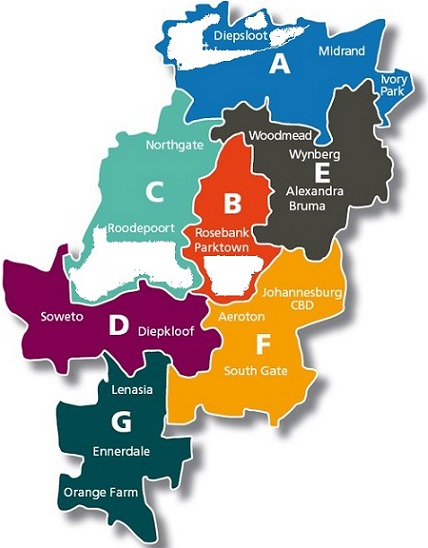
Cascade of HIV care among presumptive TB patients registered during January-June 2017 in public health facilities of Masvingo district, Zimbabwe map showing the administrative Regions of Johannesburg and the sites where the study was conducted, August to November 2015

**Study setting:** Johannesburg, the largest city in South Africa, has a population size of 4,4 million [13]. It has seven culturally and socio-economically diverse administrative regions (A-G). In 2011 region A had a population of around 609,396, region B 337,053 and region C 384,858 [13, 14]. Two public hospitals, Helen Joseph and Rahima Moosa Mother and Child Hospitals, situated in Region B, are involved in the NICD laboratory-based surveillance program [5] and were initiated as pneumonia surveillance sites in 2014 (referred to hereafter as sentinel hospitals). The survey was conducted in specific suburbs of regions A, B and C, which are catchment areas for these hospitals (Figure 1). The chosen suburbs, based on utilisation of sentinel sites, were mainly low-income areas within these regions.

**Sample size:** the sample size was calculated using Epi-Info 7 statistical software. It was calculated for pneumonia, the least common respiratory endpoint investigated in this study. Calculations were based on the assumption that 50% of total pneumonia cases would seek healthcare at sentinel surveillance sites [15]. This care seeking rate with a 95% confidence interval (CI) and a 10% precision yielded a minimum sample size of 96 without considering clustering effects. A cumulative incidence of 1% for pneumonia [16] required sampling of 9600 individuals to identify 96 individuals who had experienced pneumonia. Assuming an average household size of 3.5 translated into a minimum of 2743 households and an estimated 33% non-response rate gave a final sample size of 3650 households [17, 18].

**Sampling methodology:** a list of all enumerated households in the catchment areas of the two sentinel hospitals was acquired from 2011 census data [19]. Approximately 350 sub-places (suburbs with their extensions) were present in the three regions. Based on address information collected from sentinel sites, patients from 112 suburbs sought care at sentinel hospitals, but some were uncommon. Twenty-seven suburbs, most utilised by the sentinel sites were selected using purposive sampling. A list of households from suburbs of interest were randomly selected using STATA version 13 (StataCorp, College Station, Texas, USA). Mobile computing devices were used to access Google maps [20] to navigate to selected households.

**Definitions:** case definitions and recall periods for syndromes were based on previous healthcare utilisation surveys for comparability of data [6, 8, 9, 15, 21, 22]. ILI was defined as a sudden onset of self-reported fever or a measured temperature of >38ºC with cough and/or sore throat experienced in the last 30 days [9]. Pneumonia was defined as a sudden onset of self-reported fever or worsening fever >38ºC, cough and difficulty breathing that lasted 2-30 days, or diagnosed by a healthcare worker in the last year [23, 24]. Tuberculosis or chronic febrile respiratory illness (referred to as “tuberculosis”) was defined as fever and cough and either difficulty breathing or weight loss that lasted ≥30 days in the last year [25]. Meningitis was defined by fever and ≥2 of the following: headache, stiff neck, confusion/altered consciousness, sensitivity to bright light or seizures/convulsions; or diagnosed by a healthcare worker, within the last year. Diarrhoea defined by ≥3 loose or watery stools within a 24 hour period within the last 14 days, was recorded for children aged <5 years. A household was a shelter in which one or more people stayed for at least one night every week excluding guests and temporary visitors. A household head or responsible person was the adult person responsible for making healthcare decisions for people in the household. Seeking healthcare was defined as presenting for treatment at any healthcare facility or provider. Registered healthcare providers were defined as those with training to diagnose respiratory illnesses and examine patients at hospitals or clinics (general practitioners, nurses), while unregistered healthcare providers were defined as those with no formal training to clinically diagnose respiratory illnesses and/or examine patients (pharmacists, religious leaders and traditional healers).

**Data collection:** dedicated, trained fieldworkers collected data using a two part structured questionnaire. The household head/responsible adult was interviewed first regarding household demographics in part one of the questionnaire after giving written consent. Part one collected information on household demographics, socioeconomic status, and identified household members who reported one of the syndromes under investigation. Part two collected individual information on specific syndromes, specific healthcare utilisation, impact and expense of the illness and deaths in the household in the last year. Part two was completed following verbal consent, either by an adult with any of the syndromes under investigation or the household head. The household head could answer individual questionnaires on behalf of other household members who experienced any of the syndromes under investigation, if the relevant individuals were not at home, if they were too young or if they indicated that the head could answer these questions on their behalf. The household head was questioned regarding any household member who died within the last year. Teams visited households up to three times on separate days and times if no adult members were home on the first visit.

**Data analysis:** survey data were captured online using REDCap software [26] and analysis was conducted using STATA version 13. Proportions were calculated for demographic data and a Fisher's exact or Chi-square test was used to determine the association of characteristics among different regions. A p-value of <0.05 was considered statistically significant. Proportions with 95% confidence intervals accounting for within household clustering were calculated by syndrome type reported and healthcare facilities attended for healthcare. We calculated the proportion of people who delayed seeking healthcare and median number of days missed from work/school. The Kruskal-Wallis test was used to determine whether there were statistically significant differences between median days missed due to respiratory syndromes.

**Ethical considerations:** to participate the household head/main respondent, aged ≥18 years, was asked to sign a consent form on behalf of the household. Approval for the main survey was obtained from the Human Research Ethics Committee of the University of the Witwatersrand (study number M120367). Permission to conduct the analysis for this paper was granted by the Faculty of Health Sciences Research Ethics Committee of the University of Pretoria (study number 549/2015).

## Results

**Figure 2:**
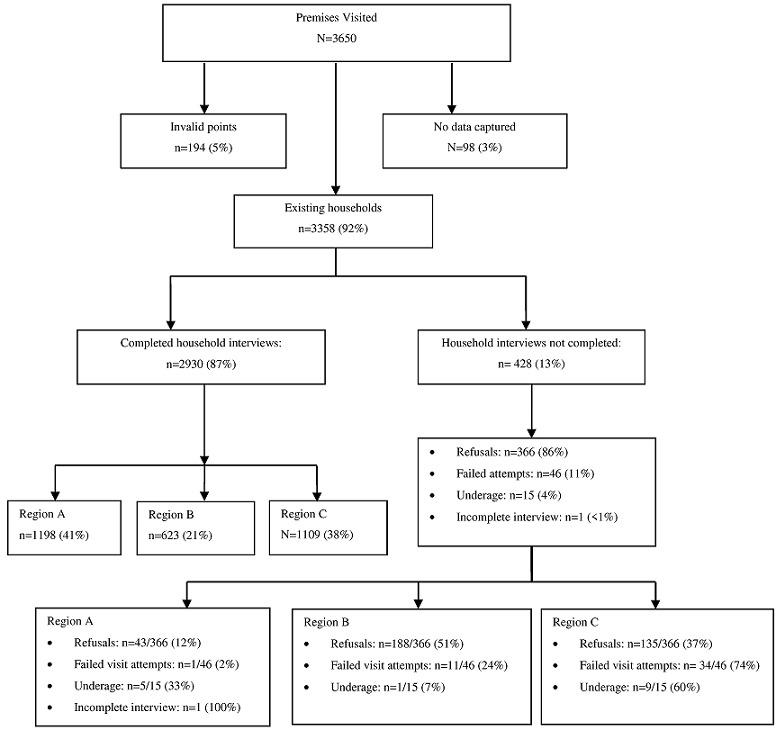
Flowchart showing the number of households enrolled in the healthcare utilisation survey conducted in region A, B and C, Johannesburg, South Africa, August to November 2015

**Participants:** of the total 3650 coordinates selected, 3358 were valid households and data were collected from 2930 (87%) of these households with 9850 individuals. This was above our estimated minimum sample size of 9600. The response rates in region A, 96%, (1198/1247), and C, 86% (1109/1291), were high, while only 76% (623/820) of households in region B agreed to participate. Of the households who did not complete interviews, most were due to refusal to participate (86%, 366/428) and half of these refusals (51%, 188/366) were from region B (Figure 2). Details were not collected with regards to reasons for refusal. Non-completions, due to no-one being home during any of the three visits (46/428, 11%), were mostly from region C (74%, 34/46).

**Demographic characteristics:** in the three regions, only half of the participants interviewed were heads of households, with the remainder of participants predominantly being relatives of the household head (Table 1). The racial composition of heads of households in the three regions differed significantly (p<0.001) with heads of households in region A 99% (718/725) and C 96% (526/547) being mainly black as compared to region B with more mixed race household heads, 41% (129/312). Education also differed between regions (p<0.001) with region A reporting the lowest percentage of household heads with a tertiary education, 3% (23/725), as compared to region B (28%, 87/312) and C (14%, 78/547). There was a difference in housing infrastructure by region with a higher proportion of informal structures (metal/tin or wood) in region A (69%, 824/1198) as compared to more formal houses (cement/brickstone) in region B (89%, 553/623) and C (73%, 815/1109) (p<0.001). In addition there was higher overall income and house ownership in Region B and C than Region A (Table 1). In terms of individual household members, just over half were female in all regions and median number of household members differed (p<0.001) (Table 2). HIV infection was reported for 3% (118/3403) of household members in region A, 1% (25/2375) in region B and 2% (98/4072) in region C (p<0.001). Among women of reproductive age (15-49 years old), a current pregnancy was reported in 5% (55/1146) of these women in region A, 2% (15/689) in region B and 3% (36/1238) in region C (p=0.001).

**Table 1:** Characteristics of households, by region, Johannesburg, August to November 2015

Characteristics	Region A n (%)	Region B n (%)	Region C n (%)	P-value
Respondent information	N=1198	N=623	N=1109	-
**Participants**				
Female	741 (61.9)	390 (62.6)	744 (67.1)	0.023
Age(years)				<0.001
18-24	216 (18.0)	107 (17.2)	202 (18.2)	
25-49	845 (70.5)	292 (46.9)	706 (63.7)	
50-90	136 (11.4)	224 (36.0)	201 (18.1)	
Unknown	1 (0.1)	0 (0)	0 (0)	
Median age in years (range)	35 (18-82)	43 (18-90)	37 (18-88)	<0.001
**Relationship to the head of household**				<0.001
Head of household	725 (60.5)	312 (50.1)	547 (49.3)	
Spouse	307 (25.6)	128 (20.5)	307 (27.7)	
Child	91 (7.6)	93 (14.9)	147 (13.3)	
Sibling	36 (3.0)	21 (3.4)	47 (4.2)	
Relatives	26 (2.2)	28 (4.5)	31 (2.8)	
Other	13 (1.1)	41 (6.6)	30 (2.7)	
Head of household information	N=725	N=312	N=547	
**Race**				<0.001
Black	718 (99.0)	103 (33.0)	526 (96.2)	
White	3 (0.4)	55 (17.7)	12 (2.2)	
Mixed race	2 (0.3)	129 (41.3)	8 (1.4)	
Asian	2 (0.3)	25 (8.0)	1 (0.2)	
**Education**				<0.001
University/College	23 (3.2)	87 (27.9)	78 (14.3)	
Grade 12	203 (28.0)	83 (26.6)	150 (27.4)	
Some secondary	339 (46.8)	115 (36.9)	205 (37.5)	
Completed primary	26 (3.6)	3 (1.0)	33 (6.0)	
Some primary	86 (11.9)	15 (4.8)	45 (8.2)	
No school	45 (6.2)	5 (1.6)	31 (5.7)	
Don’t know	3 (0.4)	4 (1.3)	5 (0.9)	
Household information	N=1198	N= 623	N=1109	
**Building material**				<0.001
Cement/ brickstone	374 (31.2)	553 (88.8)	815 (73.5)	
Wood	21 (1.8)	17 (2.7)	9 (0.8)	
Metal/tin	803 (67.0)	53 (8.5)	285 (25.7)	
House ownership				<0.001
Owned	843 (70.4)	467 (75.0)	806 (72.7)	
Renting	352 (29.3)	147 (23.6)	302 (27.2)	
Other	3 (0.3)	9 (1.4)	1 (0.1)	
**Monthly income**				<0.001
Less than R500	94 (7.8)	17 (2.7)	36 (3.2)	
R500 - R5000	873 (72.9)	247 (39.6)	552 (49.8)	
R5000 - R15000	102 (8.5)	96 (15.4)	206 (18.6)	
> R15000	6 (0.5)	68 (10.9)	49 (4.4)	
Unknown	123 (10.3)	195 (31.3)	266 (24.0)	

**Table 2:** Characteristics of individuals in households, by region, Johannesburg, August to November 2015

Characteristics	Region A n (%)	Region B n (%)	Region C n (%)	P-value
Individual household members	N=3403	N=2375	N=4072	
Median household members (Range)	3 (1-21)	5 (1-12)	4 (1-17)	<0.001
**Sex**				0.306
Female	1736 (51.0)	1259 (53.0)	2121 (52.1)	
**Age**				<0.001
<2	150 (4.4)	61 (2.6)	200 (4.9)	
2-4	252 (7.4)	132 (5.6)	283 (6.9)	
5-17	613 (18.0)	446 (18.8)	872 (21.4)	
18-64	2330 (68.5)	1561 (65.7)	2596 (63.8)	
65+	53 (1.6)	162 (6.8)	89 (2.2)	
Unknown	5 (0.1)	13 (0.5)	32 (0.8)	
**Underlying conditions**				
HIV				<0.001
Yes	118 (3.5)	25 (1.1)	98 (2.4)	
No	3053 (89.7)	2323 (97.8)	3518 (86.4)	
Unknown	232 (6.8)	27 (1.1)	456 (11.2)	
Pregnancy				0.001
Yes	55/1146 (4.8)	15/689 (2.2)	36/1238 (2.9)	
No	1085/1146 (94.7)	665/689 (96.5)	1193/1238 (96.4)	
Unknown	6/1146 (0.5)	9/689 (1.3)	9/1238 (0.7)	
Syndromes*				
Influenza-like illness	192 (5.6)	107 (4.5)	132 (3.2)	<0.001
Pneumonia	5 (0.1)	9 (0.4)	6 (0.1)	0.092
Tuberculosis	11 (0.3)	1 (0.04)	8 (0.2)	0.065
Meningitis	4 (0.1)	1 (0.04)	1 (0.02)	0.24
Diarrhoea	3/402 (0.7)	1/193 (0.5)	4/483 (0.8)	0.91
**Mortality**				0.021
Died	11 (0.3)	5 (0.2)	26 (0.6)	

* Influenza-like illness was reported in the last 30 days. Pneumonia and tuberculosis was reported for the prior year. Meningitis was reported within the last year. Diarrhoea was recorded within the last 14 days

**Frequency of reported respiratory syndromes:** five percent (466/9850) of household members interviewed reported one of the respiratory syndromes (ILI, pneumonia, tuberculosis) in the defined period (Table 2) and over 90% of these interviews were completed with the person reporting the illness when they were an adult. Of individuals reporting respiratory syndromes, 92% (431/466) reported ILI symptoms within the prior 30 days, while equal numbers of participants (4%, 20/466) reported symptoms meeting pneumonia or tuberculosis case definitions within the preceding year; very few (<1%) reported more than one syndrome, i.e. either both ILI and pneumonia (3/466) or ILI and tuberculosis (2/466). The highest proportion of individuals reporting ILI was in region A (6%, 192/3403) and the lowest in region C (3%, 132/4072, p<0.001). Pneumonia and tuberculosis cases reported among all regions totalled less than 1% and there was no significant difference among regions (Table 2).

**Frequency of other reported syndromes:** there were six reported cases of meningitis (Table 2), one in a child of 14 years and the remainder in adults over 18 years of age. Most cases were from region A (4/6, 67%). Less than 1% (8/1078) of children <5 years were reported to have experienced diarrhoea in the last two weeks (Table 2) and these were predominantly from the two regions with the highest numbers of young children, region A (3/402, 0.7%) and C (4/483, 0.8%).

**Healthcare utilisation behaviour:** sixty-eight percent (295/431) of participants who reported ILI sought healthcare and two-thirds (197/295) of those who sought care utilised registered healthcare providers (Table 3). All 20 participants who reported either pneumonia or tuberculosis sought care at registered medical providers. Primary healthcare centre consultations were similar for ILI (p=0.27) between the three regions: region A 80% (56/70, 95% CI 69-89%), region B 50% (24/48, 95% CI 35-65%) and region C 62% (49/79, 95% CI 50-73%). For all syndromes, 10% (24/237, 95% CI 7-15%) of participants sought care at sentinel hospitals, with half (12/24) from region B. Among participants who reported pneumonia, 35% (7/20, 95% CI 15-59%) sought care at sentinel hospitals. Participants who reported tuberculosis mostly sought care at primary healthcare centres 80% (16/20, 95% CI 56-94%); of these same patients 31% (5/16, 95% CI 11-58%) also sought care at public hospitals. Of all participants with tuberculosis who sought care at a public hospitals (7/20), more than half (57%, 4/7) visited sentinel hospitals. Of participants who reported ILI, 38% (115/295) also sought care at unregistered healthcare providers. The most common healthcare provider that was utilised by these participants was a pharmacy. Overall, very few participants reported consulting traditional healers or religious leaders, with no regional differences (p=0.48) (Table 3). All participants with reported meningitis utilised registered healthcare providers for care (Table 3). Two participants initially went to primary healthcare clinics, and all sought care at public hospitals. Half of all participants (3/6) went to sentinel hospitals. The majority of children with diarrhoea (75%, 6/8) had some treatment given by healthcare providers. The most common source was primary healthcare clinics (67%, 4/6). Based on self-report sentinel hospitals would not have detected 93% (184/197) of ILI cases, 65% (13/20) of pneumonia cases, 80% (16/20) of tuberculosis cases, 50% (3/6) of meningitis cases and 100% (5/5) of diarrhoeal cases.

**Table 3:** Registered and unregistered healthcare facilities that were reported to have been consulted by survey participants by syndrome in regions A, B and C, Johannesburg, August to November 2015

Healthcare providers	Influenza-like illness (ILI) * n/N % (95% confidence interval)			Pneumonia* n/N % (95% confidence interval)			Tuberculosis* n/N % (95% confidence interval)			Meningitis* n/N % (95% confidence interval)			Diarrhoea n/N % (95% confidence interval)		
	Region A	Region B	Region C	Region A	Region B	Region C	Region A	Region B	Region C	Region A	Region B	Region C	Region A	Region B	Region C
**All providers†**	110	76	109	5	9	6	11	1	8	4	1	1	1	1	4
**Registered healthcare providers**	70/197	48/197	79/197	5/20	9/20 (45)	6/20 (30)	11/20	1/20	8/20	4/6	1/6	1/6	0	1/5	4/5
	36 (29-43)	24 (19-31)	40 (33-47)	25 (9-49)	45 (23-69)	30 (12-54)	55 (32-77)	5 (0.1-25)	40 (19-64)	67 (22-96)	17 (0.4-64)	17 (0.4-64)		20 (1-72)	80 (28-99)
**Sentinel hospitals**	2/70	5/48	6/79	0	6/9	1/6	0	1/1	3/8	2/4	1/1	0	0	0	0
	3 (0.3-10)	10 (4-23)	8 (3-16)		67 (30-93)	17 (0.4-64)		100 (3-100)	38 (9-76)	50 (7-93)	100 (3-100)	0	0	0	0
**Other public hospitals**	2/70	1/48	1/79 (1)	2/5	0	3/6	2/11	0	1/8 (13)	2/4	0	1/1	0	0	0
	3 (0.3-10)	2 (0.1-11)	1 (0.1-7)	40 (5-85)	0	50 (12-88)	18 (2-52)		13 (0.3-53)	50 (7-93)	0	100 (3-100)	0	0	0
**Primary healthcare clinics**	56/70	24/48 (50)	49/79	2/5	0	1/6	9/11	1/1	6/8 (75)	2/4	0	0	0	1/1	3/4
	80 (69-89)	50 (35-65)	62 (50-73)	40 (0.4-64)	0	17 (0.4-64)	82 (48-98)	100 (3-100)	75 (35-97)	50 (7-93)				100 (3-100)	75 (19-99)
**Private hospitals**	0	1/48 (2)	3/79 (4)	1/5	3/9	1/6	0	0	0	0	0	0	0	0	1/4
	0	2.1 (0.1-11)	4 (1-11)	20 (1-72)	33 (8-70)	17 (0.4-64)	0	0	0	0	0	0	0	0	25 (1-81)
**Private clinics**	12/70	19/48	25/79	0	0	1/6	0	0	1/8	0	0	0	1/1	0	0
	17 (9-28)	40 (26-55)	32 (22-43)	0	0	17 (0.4-64)	0	0	13 (0.3-53)	0	0	0	100 (3-100)	0	0
**Unregistered healthcare providers**	45/115	30/115	40/115	0	0	0	0	0	1/20	0	0	0	1/1	0	0
	39 (30-49)	26 (18-35)	35 (26-44)	0	0	0	0	0	5 (0.1-25)	0	0	0	100 (3-100)	0	0
**Pharmacy**	42/45	30/30	35/40	0	0	0	0	0	1/8	0	0	0	0	0	0
	93 (82-99)	100 (88-100)	88 (73-96)	0	0	0	0	0	13 (0.3-53)	0	0	0	0	0	0
**Traditional healers**	2/45	0	0	0	0	0	0	0	0	0	0	0	0	0	0
	4 (1-15)	0	0	0	0	0	0	0	0	0	0	0	0	0	0
**Religious leaders**	1/45	1/30	5/40	0	0	0	0	0	0	0	0	0	0	0	0
	2 (0.1-12)	3 (0.1-17)	13 (4-27)	0	0	0	0	0	0	0	0	0	0	0	0

* Some participants sought care at more than one facility; †People who did not seek care for ILI 32% (141/431); †Children who did not receive care for diarrhoea 25% (2/8)

**Delay in seeking healthcare:** a delay in seeking healthcare, i.e. accessing care three or more days after symptom onset, was reported by 26% (75/295) of the participants who reported ILI, 35% (7/20) of those who reported pneumonia and 60% (12/20) of participants who reported tuberculosis. Four participants who reported meningitis and only one child with diarrhoea had a delay in accessing healthcare. Participants stated “not feeling sick enough” as the most common reason for delaying seeking healthcare for all syndromes, while worsening of symptoms and symptoms that were not getting better were the main reasons for deciding to present at healthcare facilities for care. Less than 10 patients in total reported cost, travel time or waiting time as a reason for delaying care.

**Absenteeism due to different syndromes:** one third of participants (155/466) with any respiratory syndrome reported missing school or work because of illness. The median number of days missed differed significantly between the three respiratory syndromes (p<0.05). Participants with ILI reported missing a median (range) of 2 days (1-14 days); those with pneumonia 13 days (1-30 days) and those with tuberculosis 5 days (1-30 days) per episode. Patients who reported meningitis generally missed extended periods of school/work because of illness or long hospitalisations; median of 30 days (3-60 days).

**Mortality:** a total of 42 individuals were reported to have died in the year prior to the survey; 11 (0.3%) in region A, 5 (0.2%) in region B and 26 (0.6%) in region C. Two deaths were in children <5 years of age; both had death certificates and one died in a sentinel hospital. Of the remaining deaths in older individuals, 58% (23/40) died in hospital (5 in a sentinel hospital) and a similar proportion (23/40) had death certificates. The causes listed on the death certificates that were viewed (n=24) in all age groups included, natural causes (11), unnatural causes (3), respiratory causes (4), cardiac causes (3), diabetes (1), cancer (1) and meningitis (1).

## Discussion

In this study, we describe health-seeking behaviour for common infectious syndromes, including three respiratory syndromes, meningitis and diarrhoea, in three regions of Johannesburg, South Africa. This study identified rates of infectious diseases similar to previous surveys [21, 22], and most reported disease was mild. We identified that health seeking behaviour differed by syndrome type and region and that severity of illness usually prompted individuals to seek healthcare. Our study showed that sentinel sites were most likely to detect sicker patients from certain areas. Overall, the two sentinel surveillance hospitals were mainly utilised by participants in regions B and C whose syndromes were severe. This could have been due to ease of access as regions B and C were in close proximity to the hospitals. Even though distance to healthcare provider was not stated as a main barrier in the study, a previous study in South Africa reported distance to healthcare facility as a barrier [27]. The catchment area was broad and not all areas were covered. This could have biased the estimated use of sentinel hospitals. ILI was the most common syndrome reported in this study at 4%. In South Africa, a typical influenza season occurs during the Southern Hemisphere's winter months (May-August) [28]. The survey was conducted at the end of the influenza season. The survey timing and shorter recall period for ILI (30 days) could have resulted in a biased estimate that underestimated ILI prevalence. A similar prevalence of ILI (4.9%) was reported in another HUS conducted from October to December 2013 in South Africa [21] with slightly higher prevalence (>6%) reported in surveys conducted in Kenya [6, 15]. As part of the ILI surveillance program in South Africa (May 2012-April 2016) the detection rate of influenza in patients with ILI was 13.9% (666/4797) [3]. Less than 1% of individuals in our study reported experiencing pneumonia or tuberculosis syndromes; these results are similar to those obtained for a South African HUS conducted in 2013 [21], but slightly lower than the 2% prevalence for pneumonia in another HUS from 2012 [22]. A lower prevalence of tuberculosis and pneumonia syndromes compared to ILI is expected as these syndromes are less common. The prevalence of reported meningitis cases was lower than reported in our previous surveys, possibly due to a more specific case definition [21, 22]. In all three regions, participants who reported ILI mainly sought healthcare at primary healthcare clinics and pharmacies. Only a few went to traditional healers and religious leaders for care. These findings are supported by a general household survey conducted by Statistics South Africa which identified primary healthcare clinics as the first point of care for mild illness and that traditional healers were not commonly consulted [18]. The majority of participants who reported pneumonia or meningitis sought healthcare at public hospitals, with most seeking healthcare at the sentinel sites. In our study, people were more likely to seek healthcare only after experiencing severe symptoms. The South African healthcare system is structured in such a way that primary healthcare clinics are the entry point for seeking care; people present at clinics with mild ailments and are only referred to hospital when their symptoms are severe. In other surveys, participants who reported pneumonia mainly sought care at hospitals [9, 15]. Another study reported hospitals as point of care for pneumonia [23] and this was linked to illness severity. Tuberculosis diagnosis and treatment in South Africa has been rolled out at primary healthcare facilities; this could explain why most people who reported tuberculosis sought care at these clinics.

Early disease treatment plays a role in reducing morbidity and mortality [29, 30]. Just over half of study participants indicated that not feeling sick enough was the main reason they delayed presenting to facilities for healthcare. These findings are consistent with other studies, including a national survey in South Africa, which reported not being sick enough as the main reason people delayed seeking healthcare regardless of syndrome [15, 31, 32]. On the contrary, another study conducted in rural and urban areas of South Africa stated personal finances and distance to healthcare facilities as the main reasons for delaying seeking healthcare [12, 33]. Participants in our study stated worsening symptoms as the main reason for eventually deciding to seek healthcare and this correlated with another South African study [12]. Missing school or work due to respiratory illness was frequently reported. The more severe the syndrome, the more days were missed. Being absent from school or work has negative social and economic implications. Studies conducted in high-income countries reported that absence from work/school due to severe ILI resulted in loss of productivity [34, 35]. Our study had some limitations. First, although we used standard survey case definitions, which makes our data comparable, it does not rule out the possibility of overestimating ILI because of low specificity of the case definition. Interviewers did not have medical training, which could have resulted in some misclassification between acute and chronic and mild and severe respiratory disease. Second, data collected on all syndromes were based on participant self-report and recall with no laboratory confirmation or medical record review. The recall period differed between syndromes, which may have led to differential reporting and underreporting of syndromes with a longer recall period. Other studies have shown that shorter recall periods may reduce bias [7]. Our results were not dissimilar to previous surveys which may have been subject to similar recall bias [21, 22]. Third, a proportion (12%, 58/485) of interviews were conducted with household members other than those reporting individual illness. This may have affected data completeness for individual illnesses and underreporting of underlying conditions such as HIV. In this study, the HIV prevalence was 2-5% while the estimated South African national HIV prevalence in 2012 was 12.2% [36]. The reported pregnancy rate (3.4%) was lower than that reported in the 2015 household survey (5.3%), but the latter included pregnancies in the last 12 months, while we only recorded current pregnancies [18]. Fourth, more households refused participation in region B; this may have biased the representativeness of our findings as region B had a different socio-economic profile. Region B was economically more affluent than other regions and socio-demographic, racial and economic factors have previously been associated with willingness to respond to surveys [37], which may account for the low-response rate in this region. A higher proportion of individuals from Region B accessed private healthcare and were less likely to attend sentinel hospitals for care. Individuals who feel that a survey would benefit them in some way are more likely to respond which could introduce response bias. Fifth, we did not cover all areas served by the sentinel hospitals, which may have resulted in a biased estimate of sentinel site use. Sixth, we only achieved about 20% of our target sample size for pneumonia which limited our ability to conduct sub-analyses. Last, while we made an effort to represent all residential areas, including informal settlements, it is possible that new settlements, transient populations or those living in non-residential areas, such as commercial districts, may have been systematically excluded from the sample. The strengths of this study include that households were randomly chosen using a list of all enumerated households from Statistics South Africa, the overall response rate was high and teams were monitored to ensure that completion of all syndrome information was standardised. In addition suburbs that were chosen were those identified from the surveillance program to be the source of the majority of patients attending the sentinel sites. The age structure of households was similar to that reported in the national census [19], with 11% of members <5 years and only 3% >64 years of age.

## Conclusion

This study highlights healthcare seeking patterns within the catchment areas of a hospital-based pneumonia and laboratory-based surveillance program. Of patients with pneumonia and meningitis who sought care at public hospitals, at least half sought care at sentinel hospitals; this indicates surveillance program relevance in capturing severe disease syndromes, whereas those with milder syndromes mainly sought care at public health clinics. Using HUS data in conjunction with surveillance data will assist in estimating a more precise burden of these syndromes and aid in allocation of resources.

### What is known about this topic

- In South Africa, public government healthcare facilities are commonly consulted for infectious syndromes;
- Different barriers to healthcare exist.

### What this study adds

- Describes healthcare-seeking patterns providing context for surveillance data from public healthcare facilities;
- Identifies barriers to healthcare access among residents of public hospital catchment areas;
- Allows more accurate estimation of the burden of infectious respiratory illnesses to improve targeting of prevention and treatment strategies in these communities.

## Competing interests

The authors declare no competing interests.
